# Automatically detecting trends and open questions from mental health publications: a Wellcome-funded GALENOS project

**DOI:** 10.1136/bmjment-2025-302379

**Published:** 2026-04-02

**Authors:** Janna Hastings, Marie Wosny, Jaycee Kennett, Ava Homiar, Gin S Malhi, Toshi A Furukawa, Jennifer Potts, James Thomas, Andrea Cipriani

**Affiliations:** 1 Human-Centered Health AI, Idiap Research Institute, Martigny, Switzerland; 2 Swiss Institute of Bioinformatics, Lausanne, Switzerland; 3 School of Medicine, University of St Gallen, St Gallen, Switzerland; 4 EPPI Centre, UCL Social Research Institute, University College London, London, UK; 5 Department of Psychiatry, University of Oxford, Oxford, UK; 6 Division of Clinical Informatics, Harvard Medical School, Boston, Massachusetts, USA; 7 Academic Department of Psychiatry, Kolling Institute, Northern Clinical School, Faculty of Medicine and Health, University of Sydney, Sydney, New South Wales, Australia; 8 Kyoto University Office of Institutional Advancement and Communications, Kyoto, Japan; 9 Oxford Precision Psychiatry Lab, NIHR Oxford Health Biomedical Research Centre, Oxford, UK; 10 NIHR Oxford Health Clinical Research Facility, Oxford Health NHS Foundation Trust, Warneford Hospital, Oxford, UK

**Keywords:** Anxiety Disorders, Depressive Disorder, Psychotic Disorders

## Abstract

**Background:**

More effective and better tolerated treatments are urgently needed for people with mental health disorders, such as anxiety, depression and psychosis. However, the rate of translation of positive results from early phase studies into clinically validated treatments remains painstakingly slow. The scientific literature on mental health preclinical and early interventions is burgeoning at pace, making it difficult for researchers, practitioners and policymakers to identify and track new developments.

**Objective:**

As part of the Wellcome-funded Global Alliance of Living Evidence for aNxiety, depressiOn and pSychosis project, we aimed to develop and evaluate an automated approach to track the evolution of mental health research over time, detect emerging trends and suggest open questions.

**Methods:**

Our approach used topic modelling, large language models and time-series forecasting in combination. We applied our approach to a corpus of 182 747 titles and abstracts extracted from the OpenAlex database for 2015–2025. Using topic modelling to identify topics and then tracking topic mentions over time, we built a time series predictive model and predicted ‘trendiness’ based on sustained increased mentions above baseline expected from model predictions. We evaluated our approach retrospectively using a blinded expert study of a randomly selected sample of trending and not trending topics. Finally, we developed a novel topic-augmented generation approach to suggest open questions in trendy topics and evaluated the approach by comparison to baseline-generated questions without topic augmentation.

**Findings:**

Our approach detected 973 topics and predicted 165 (17%) of those as trending. Key topics that the model predicted as trending included ‘ketamine for treatment-resistant depression’, ‘student mental health in academia’ and ‘COVID-19 psychosis’. We found that domain experts largely agreed with the model’s predictions of trendiness. Topic-augmented generated questions were more specific than baseline generated questions.

**Conclusions:**

Our approach enables identification of new developments and open questions. Future work will improve temporal pattern tracking and use full texts.

**Clinical implications:**

Our approach can support all stakeholders to gain an overview of the published literature, assess temporal patterns, identify trends and rank open questions.

**WHAT IS ALREADY KNOWN ON THIS TOPIC**

- The mental health research literature is expanding rapidly, making it increasingly difficult for researchers, clinicians and policymakers to identify emerging therapeutic developments and research priorities in a timely manner.

**WHAT THIS STUDY ADDS**

- This study demonstrates that combining topic modelling, large language models and time-series forecasting can automatically detect trending research areas (such as ketamine for treatment-resistant depression) and generate relevant open questions, with predictions validated by domain experts.

**HOW THIS STUDY MIGHT AFFECT RESEARCH, PRACTICE OR POLICY**

- Our approach to automated research surveillance could help stakeholders systematically monitor the mental health research landscape, prioritise emerging treatment targets and accelerate the translation of promising interventions from early studies to clinical practice.

## Background

 Despite significant investment in mental health, the burden of psychiatric conditions globally remains high.^1 2^ New treatments and early interventions are urgently needed, yet there have been few novel therapies that have made a meaningful difference to clinical practice in recent years.^3^ Early preclinical findings can lead to promising new directions for the development of novel treatments and for better understanding of individual effects of current treatments, but it is difficult to gain an overview of recent findings and the rate of translation remains unacceptably slow.^4^ One contributing factor is that the body of published research on mental health (both preclinical and clinical) is vast and fast-growing and difficult to piece together, as it is fragmented across many different journals and publications.^5^ Nearly half a million relevant articles are found in the PubMed^6^ literature database over the last decade mentioning at least one of the key terms ‘anxiety’, ‘depression’ or ‘psychosis’ (not considering synonyms), and the annual publication rate is increasing^7^ with growth averaging 7% per year (online supplemental table S1). This creates challenges for clinical researchers, practitioners, policymakers, research funders and other stakeholders to keep up with all the published research in the field.^8^

The Wellcome-funded Global Alliance for Living Evidence on aNxiety, depressiOn and pSychosis (GALENOS) project aims to systematically map and synthesise the research literature to identify key preclinical advances, better understand mechanisms and advance diagnosis and treatment, by providing systematic reviews on topics of relevance.^5 9^ Evidence synthesis in systematic reviews is the process of systematically integrating published evidence in order to answer specific questions based on integrated and potentially heterogeneous evidence sources.^8 10^ To date, the GALENOS project has completed several key systematic reviews on a diverse range of topics including the role of trace amine-associated receptor 1 agonism in psychosis,^11^ the effect of exercise on the effectiveness of psychotherapy in post-traumatic stress disorder^12^ and the efficacy of pro-dopaminergic pharmacological interventions for anhedonia in depression.^13^ Identifying trends and open questions in publications on mental health is essential for surveying emerging literature in fields where the evidence is rapidly evolving. Identifying topics that show a pattern of increasing interest over time, and generating potential associated research questions, can be used to inform priority setting. However, such overviews do not yet exist in general.^8^ As an added challenge, in mental health research, there are large differences in theoretical frameworks and vocabularies, and further, reporting and publication practices across different subdisciplines can vary.^5 14 15^ Siloed thinking of experts from differing disciplines risks guiding topic choices and research questions in systematic reviews in ways that may limit treatment choice options.^16^

Computational approaches to automate text processing are increasingly being used in mental health research.^17 18^ Recent advances in artificial intelligence, including large language models (LLMs) and related technologies, have created a step change in automation possibilities to derive insights from published literature.^19 20^ Automated approaches to detect topics in text are enhanced by the flexible and contextual ‘embeddings’ of text provided by LLMs, leading to richer and more coherent topics.^21^ Previous work has developed approaches to detecting, tracking and predicting publication trends of gene mentions^22^ and of news topics.^23^ However, in these approaches, the topics were defined by a small number of key words or phrases (eg, gene or politician names), which is different to in the mental health literature where topics and themes reflect quite subtle combinations of complex and heterogeneously described entities.

## Objective

In the present study, we aimed to develop an automated approach to mining the mental health literature to identify trending topics, including both newly emerging topics as well as pre-existing topics experiencing a recent sudden surge in interest, and evaluate our predictions using a blinded expert study. We furthermore aimed to generate open research questions within identified topics using a novel ‘topic-augmented’ generation approach, which we evaluate by comparison to a baseline of question generation without topic augmentation.

## METHODS

### Literature extraction and cleaning

All data processing and analysis were conducted in Python. We extracted data for the time period 1 January 2015 to 28 February 2025 from OpenAlex^24^ using the following search string across titles and abstracts (search date: 17 March 2025).

“(“mental” OR “psychological” OR “behavioural” OR “psychology” OR “psychiatry” OR “neurological” OR “mind” OR “brain” OR “behaviour” OR “psychiatric”) AND (“anxiety” OR “depression” OR “psychosis”) AND (“treatment” OR “therapy” OR “therapeutic” OR “mechanism” OR “intervention” OR “early” OR “diagnosis” OR “diagnostic” OR “translation”)”

This search was designed to encompass the GALENOS project’s focus on anxiety, depression and psychosis, targeting early interventions, mechanisms or diagnostic or translational innovations. The database OpenAlex was selected rather than PubMed in order to track a wider range of research publications including conference abstracts and books. For each article in the search results, title, abstract and core metadata including publication year and authors were extracted. Duplicate records were removed based on unique paper identifiers or combinations of titles and authors. Articles missing titles, abstracts, authors or publication date were excluded, and a language detection step was applied to titles and abstracts to exclude non-English articles. Online supplemental materials, corrections, tables, figures and other artefacts were removed based on keywords. Unusually short (title <3 words, abstract <80 words) or long (title >60 words, abstract >1000 words) texts were filtered out, alongside withdrawn articles and non-research content, such as editorials or letters. Text fields were cleaned to remove HTML artefacts.

### Identification and naming of topics

To identify key topics in the extracted literature, we trained a BERTopic model^25^ based on SentenceTransformer embeddings created with the ‘all-MiniLM-L6-v2’ model. For each article, title and abstract were combined. The parameters associated with the topic model, which control the number of topics, were: UMAP n_neighbors=10, n_components=15, metric='cosine’; HDBSCAN min_cluster_size=15, min_samples=15, metric='euclidean’. We removed standard English and PubMed stopwords. Each resulting topic was represented by its 15 most relevant words.

Next, we assigned detected topics to documents (ie, titles and abstracts). Considering that a given study may have different interacting topic elements, we fitted an approximate topic distribution, which assigned a probability in the range (0,1) to each topic in each document. A binary matrix was then constructed by considering a topic as mentioned in a document if the probability for that topic exceeded 0.08, which threshold was selected empirically to yield a distribution of topic counts per document that peaked at 1–2 topics per paper without exceeding 10 (online supplemental figure S1).

As topic models do not generate names for the topics they retrieve, we applied an open LLM to the lists of relevant keywords for each topic to identify a brief and meaningful title. We executed the ‘LLaMA 3.3–70 b’ model via the DeepInfra API with the prompt ‘Please give a concise word or phrase to describe the main topic that unifies the following words: {topic_words}. Please return only the word or phrase with no explanation. Topic:’ in which topic_words was replaced with the series of 15 characteristic keywords for each topic.

### Time series predictive model of topic mentions

Topic mentions in documents were aggregated over documents at weekly, monthly and annual periods, enabling the creation of binary matrices of topic mention frequencies over these time periods. The monthly period was selected as the basis for further predictions after exploring the temporal patterns of publications in the dataset (online supplemental figure S2), as there is significant noise at the weekly level due to ‘spikes’ in the dataset, while the yearly level provides too low a resolution for meaningful predictions.

We trained a Sequential time series predictive model with two layers of Gated Recurrent Units each with 10 neurons, followed by a dense output layer for the final prediction of the future number of mentions of a topic given the time series of previous mentions of that topic. As input, we used a sliding window of 6 months of consecutive topic mention counts, and as output we predict the next month’s topic mention count. The period of 6 months was selected to provide a sufficient window of prior data for model predictive performance while still enabling recent trends to be detected. The model used the *Adam optimiser* with mean squared error as loss and was trained for 10 epochs with a batch size of 32. For each topic, we trained a separate model with the data from the other topics and then used that model to make predictions for the unseen model 1 month at a time. This produced a predicted time series for each topic from 6 months onwards.

### Topic trendiness prediction and ranking

To predict emerging topics with surges in interest, at any given time point, we determine the difference between the predicted and the actual time series of the topic for *n* of the previous *m* months. A topic was regarded as trending if its actual mentions outperform predicted mentions in a sustained way over the time period, similarly to the approach followed in previous work on gene mentions.^22^ In our implementation, we consider a topic as ‘trendy’ if the actual number of mentions exceeds the predicted mentions for n=4 of the previous m=6 months. Trendiness in this sense is thus distinguished from popularity, as a topic that is consistently popular will not be considered trendy by this definition. The threshold of n=4 was selected to allow for some variations within the 6-month period, and the overall period of 6 months was selected to enable relatively recent shifts in publication practices to be detected. As these thresholds are arbitrary, we also conducted a sensitivity analysis to explore how the predictions would vary with these parameters (online supplemental figure S3A,B). We furthermore tested the sensitivity of topic trendiness prediction within the top 50 topics to potential redistribution of papers assigned to a January spike to other months within the same year across five runs, each of which adding 20% more random redistribution (online supplemental figure S3C).

After detecting that a topic is trending, we furthermore assigned a rank by calculating the magnitude of the difference between predicted count of mentions and actual count of mentions. Similarly to previous work in BERTrend,^23^ we added a decay factor to prioritise recency (more recent outperformances count more than older outperformances). We normalised the rank to the size of the topic in terms of numbers of mentions, so that the rank does not simply track the baseline size of the topic.

The resulting trendiness rank for a topic *t* is given by:


$$TR_{t}=\sum_{i=1}^{m}\left(a_{i}-p_{i}\right)e^{1/\left(m-i\right)}/(x=\frac{\sum_{i=1}^{m}a_{i}}{m}\;if\;x>1\;else\;1)$$

where *m*=number of months, *a*=actual monthly count of mentions in month *i*, and p*=*predicted count of mentions in month *i*. The rank is then normalised by dividing by the mean *x* of the actual mentions over the time period, or 1 if the mean *x* is less than 1 to avoid inflation of very-low-prevalence topics.

For the evaluation of our approach, we applied this algorithm to predict trendiness at the time point of February 2025. We evaluated our trend prediction approach through a blinded user survey study in which domain experts were asked to rate topics as to whether they were ‘trending recently’, ‘popular but not specifically trending recently’ and ‘neither popular nor trending recently’. Domain experts were recruited to respond to the survey using purposive sampling from the GALENOS international advisory board, which consists of experts in the field of mental health with an academic background in psychiatry or psychology. In total, 12 experts completed the survey.

Ten trendy topics and 10 non-trendy topics were randomly selected and arranged randomly in the survey. For each topic, participants were shown the characteristic keywords generated by the topic model, along with an exemplary paper in which the topic appeared. The survey was implemented using Google Forms and included 20 questions, each of which gave the topic name, list of characteristic words and one selected paper from the topic, before asking whether the topic was trendy, popular but not specifically trending, or neither popular nor trendy. Finally, an open-ended ‘Other’ response option allowed users to provide qualitative feedback. The survey questions are listed in full in online supplemental file 1. The trendy and not trendy topics were sorted randomly in the survey, but all experts received the questions in the same sequence. The resulting data from the survey were counted and statistically tested for association with the predicted categories of ‘trendy’ and ‘not trendy’ using χ^2^ test for categorical association.

### Deriving research synthesis questions from topics

To identify potential research synthesis questions for consideration, we prompted an LLM with the detail of extracted topic keywords and exemplary abstracts following a ‘topic-augmented’ generation paradigm. For evaluation, we compared the questions generated using this method to a baseline of ‘zero shot’ generated questions for the same topic without providing the context of the topic keywords and exemplary associated abstracts.

For this task, we used the model ‘Qwen/Qwen3-Next-80B-A3B-Instruct’ executed via the DeepInfra API. The role prompt provided for the system to follow was: ‘You are a helpful expert assistant for working with research synthesis topics from the scientific literature in the field of mental health’. The baseline instruction prompt for the zero-shot generation scenario, corresponding to the default behaviour of interacting with a language model without topic-guided specificity, was: ‘Please generate a list of 3–5 current open questions for the topic ‘{topic_name}’ where a systematic review would be helpful. Format response in JSON’. For the topic-augmented generation scenario, the instruction prompt was: ‘Please extract a list of 3–5 current open questions for the topic '{topic_name}’ identified by characteristic words '{topic_words}', where a systematic review would be helpful, from the following recent abstracts for this topic: '{abstracts}'. Format response in JSON’.

In both cases, the topic_name was replaced with the name assigned to the topic. In the second case, topic_words was additionally replaced with the 15 characteristic words for the topic extracted from the topic model, and 10 randomly selected abstracts for that topic published within 2025 were injected.

For evaluation, we executed the two approaches for the 10 trendy topics from the survey. We calculated average perplexity (a measure of how predictable a text is, lower=more predictable), lexical diversity (a measure of vocabulary richness, lower=less diverse) and information content (a measure of how specific a text is, lower=less specific) for the generated text and compared the distributions of these metrics between conditions.

## FINDINGS

### Publication and topic patterns over time

**Figure 1:**
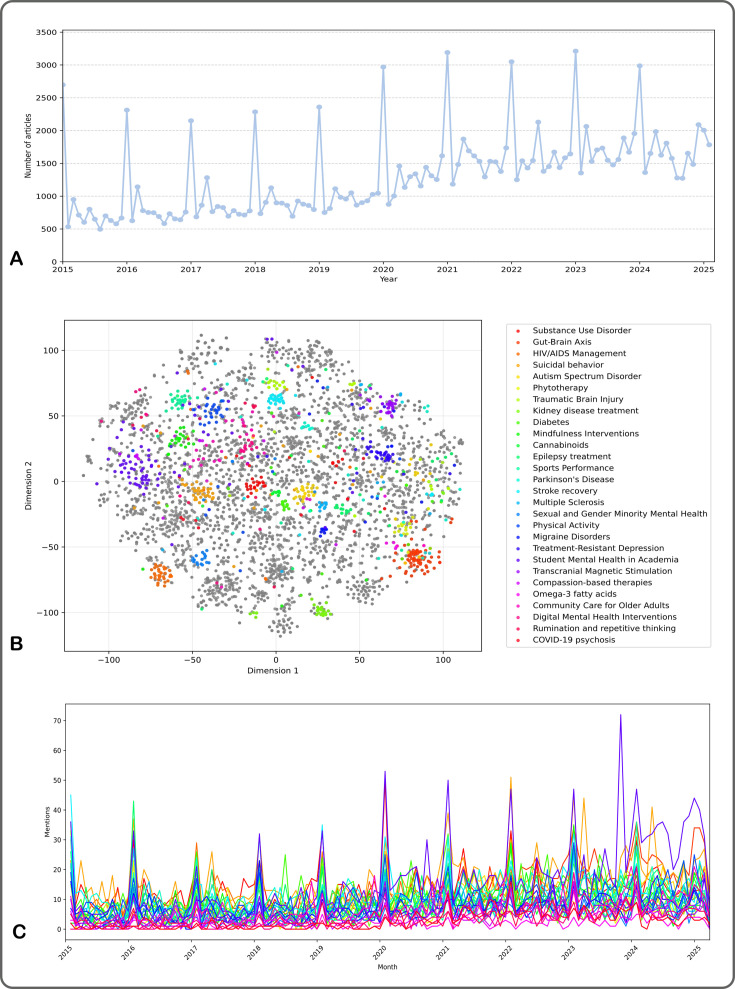
Corpus of publications and associated identified topics. (**A**) Number of publications related to anxiety, depression and psychosis per month in the dataset 2015–2025, showing January spikes. (**B**) Embedding visualisation of abstracts, coloured by selected topics. (**C**) Monthly mentions of selected topics in the abstract dataset.

The OpenAlex search returned 182 747 articles related to anxiety, depression and psychosis over the 10-year period. Of these, 159 950 (87.53%) were retained after all data cleaning and duplicate removal operations (Dataset available at Hastings et al. Data^26^). The total numbers of publications are shown per month in figure 1A. There is a noticeable overall increase in publications on our specified topics in recent years, reflecting overall publication trends. Data for 2025 were not yet complete at the time of extraction. There is also a spike in publications in January of each year, which is largely a technical artefact insofar as a publication may be reflected as belonging to January if the month of publication is not reported. The January spike introduces a temporal bias into the dataset and predictive model; however, this can be assumed to affect all topics equally.

The model identified 973 distinct topics, and each was assigned a name. The full list of topics is available as online supplemental material; a selection of topic names is illustrated in figure 1B. There is partial overlap between some topics: larger topics encompass smaller ones hierarchically (online supplemental figure S4). Although some of the topic names may appear to reflect non-mental-health-related topics, for example, kidney disease and diabetes, in fact these topics encompass the psychosocial and mental health implications of those diseases, such as anxiety in transplant patients, or comorbidity between depression and diabetes. The distribution of topic mentions over time in monthly intervals for selected topics is illustrated in figure 1C; the distribution at weekly and annual intervals is shown in online supplemental figure S2. The monthly temporal unit was selected as the most relevant unit for balancing signal versus noise for the analysis and prediction of trendiness.

### Trendiness prediction and ranking

**Figure 2:**
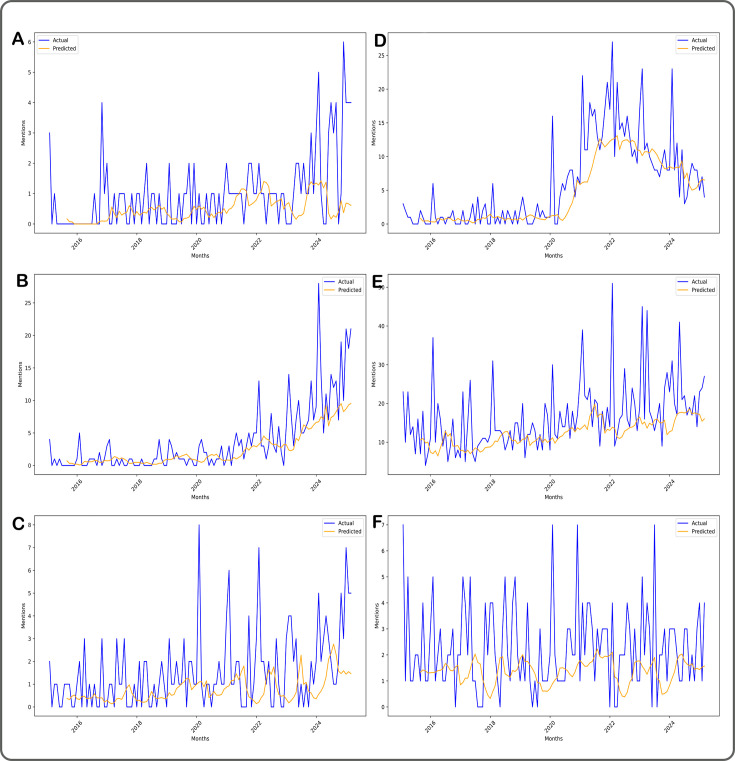
A selection of topics illustrated that are trendy (**A–C**) and not trendy (**D–E**). (**A**) Interoception, an example of a relatively low-frequency topic that nevertheless shows a clear pattern of recent interest. (**B**) Artificial intelligence applications in mental health, an example of a topic with sustained interest but also a clear pattern of growing recent interest. (**C**) Refugee mental health, showing patterns of recent trendiness alongside historical spikes and falls. For all plots, the blue line shows the actual mentions of the topic while the orange line shows the contrasting predicted mentions of topics. The y-axes have different ranges in the three plots while the x-axes all show the same monthly time points. (**D**) The mental health implications of pandemics/coronaviruses on families, showing a characteristic ‘pandemic’ pattern of clear and sustained trending throughout 2020–2022 followed by a decline in interest until the present day. (**E**) Suicidal behaviour, a topic that is clearly popular and frequently mentioned, but not specifically trending recently. (**F**) Eating disorders and brain stimulation, a topic that has both low average monthly mentions and is not trending recently.

The mean absolute error for the time series predictive models was 1.52 with SD 0.057, reflecting overall good performance. Our trendiness detection approach rated 165 out of the 973 (17%) topics as trending. Three of the top-ranked trendy topics are illustrated in figure 2A–C, reflecting different baselines of numbers of publications per month; the full list of ranked topics is available as online supplemental material. Figure 2A illustrates a topic, interoception, that has overall few mentions in the historical dataset, but has noticeably increasing interest in recent months, representing ‘topic mentions’ behaviour associated with an emerging newly trendy topic. Figure 2B illustrates a topic related to artificial intelligence in which the pattern of mentions has been steadily growing in recent years and is still on an upwards trajectory. Figure 2C shows a topic, refugee mental health, which has some prior waves of interest that receded but still a recent pattern of upward trending. Among the not trendy topics, figure 2D–F, figure 2D shows a COVID-related topic with a clear surge of interest and trendiness in the COVID time period and immediately afterwards, but reducing interest in the more recent years, figure 2E shows a suicide-related topic with a sustained high level of interest but not specifically trending recently, and figure 2F shows a topic related to eating disorders that has relatively low and stable monthly publications.

**Figure 3:**
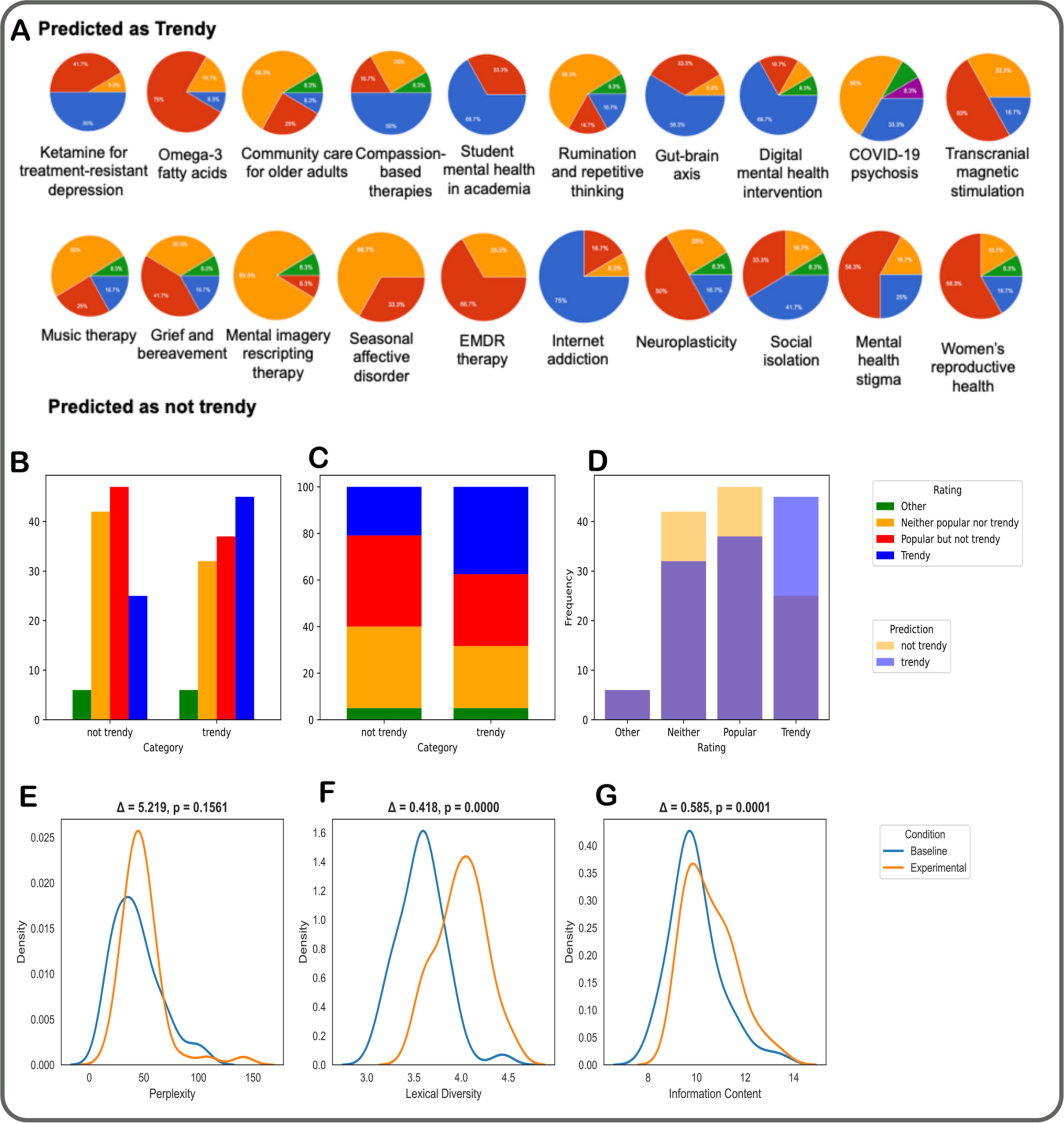
(**A**) The evaluations given by the experts for the 10 ‘predicted as trendy’ topics (top row) and the 10 ‘predicted as not-trendy’ topics (bottom row) included in the survey. For the expert ratings, blue indicates expert-assigned ‘trendy’, red indicates expert-assigned ‘popular but not specifically trending recently’, orange indicates expert-assigned ‘neither popular nor trending’ and green indicates ‘other’. (**B**) Frequency distributions of the expert ratings in the two groups of topics, predicted not trendy and predicted trendy. (**C**) Frequency percentages of the expert ratings for the two groups. (**D**) Overlapping histograms of ratings. For baseline and topic-augmented generated questions: (**E**) perplexity, (**F**) lexical diversity, (**G**) information content. EMDR=Eye Movement Desensitisation and Reprocessing.

As there is ultimately no ‘ground truth’ for which topics are trendy, we conducted an expert evaluation with the dual objective both of determining the extent to which experts agreed with our model’s predictions as well as to receive open-ended feedback to inform the approach. The full set of expert responses is illustrated in figure 3A. The survey revealed that different experts do not necessarily agree on whether a topic is trendy or not, with differing levels of agreement between topics. Overall, we saw poor agreement with a mean Cohen’s pairwise Kappa of 0.158, SD 0.184, range (−0.111–0.621) and Fleiss’s Kappa for all raters of 0.141. Despite this, treating each expert’s rating individually as a separate data point, we found that the trendy topics were rated trendy more often than the not trendy topics. As shown in figure 3B–D, the rating ‘trendy’ was selected more often for topics that were predicted as trendy than for topics not predicted as trendy (p<0.05 in χ^2^ test for categorical association). Interestingly, while for the majority of topics in the survey the expert ratings agreed at least in part with the prediction, there are some topics where experts clearly disagreed with the model while largely agreeing with each other: a topic that experts rated as not trendy and the model rated as trendy was ‘community care for older adults’, and one that the experts rated as trendy and the model not was ‘internet addiction’. Both these topics are relatively popular and the temporal pattern of their mentions revealed repeated waves of historical interest (online supplemental figure S5), highlighting the need to understand overarching publication trends beyond the most recent time.

### Deriving candidate research synthesis questions from trendy topics

We show the questions generated by the baseline and topic-augmented approaches in table 1 for three exemplary topics: ketamine for treatment-resistant depression, compassion-focused therapies and rumination and repetitive thinking. The full set of generated questions is in online supplemental table S2. We observe that instruction following performance was good: in all cases, the model generated five candidate research synthesis questions, without repetition. Interestingly, the generated questions are a mix of prognostic, treatment and mechanism-focused questions, all key strands of research in line with the GALENOS project’s aims. We can observe some similarities in the generated questions between the two scenarios, for example, for topic ‘rumination and repetitive thinking’ the first generated review question in both cases reflects on the different subtypes of rumination and different population groups. However, in most cases, the questions generated in the baseline scenario are broader and more overarching than the questions generated in the topic-augmented scenario, in line with our hypothesis. We compared the baseline and topic-augmented scenarios according to perplexity, lexical diversity and information content (figure 3E–G) and found that the experimental condition significantly outperformed the baseline in two out of three metrics.

**Table 1:** Exemplary generated research questions for systematic reviews in two conditions: baseline, prompting the model with task and topic name and topic-augmented generation, prompting the model with the task, topic name, list of key words for the topic and list of 10 exemplary recent titles and abstracts from the topic

Topic name	Baseline zero-shot generation approach	Our topic-augmented generation approach
Ketamine for treatment-resistant depression	What is the comparative effectiveness of novel neuromodulation techniques (eg, TMS, ketamine, esketamine, VNS and DBS) in reducing depressive symptoms and improving functional outcomes in patients with treatment-resistant depression across different symptom profiles and comorbidities? Among patients with treatment-resistant depression, what are the long-term (≥2 years) outcomes of pharmacological augmentation strategies (eg, lithium, thyroid hormone, atypical antipsychotics) vs switching to alternative mechanisms of action (eg, MDMA-assisted therapy, psilocybin), and how do these outcomes vary by biomarker profiles or genetic subtypes How do psychosocial interventions (eg, CBT, DBT, ACT and behavioural activation) modify treatment response when combined with pharmacological or neuromodulatory interventions in treatment-resistant depression, and which patient subgroups benefit most from integrated models What is the role of inflammatory, metabolic and gut-microbiome biomarkers in predicting resistance to standard antidepressants and guiding personalised treatment selection in treatment-resistant depression, and do interventions targeting these pathways improve clinical outcomes? How do real-world treatment sequences and adherence patterns in diverse healthcare settings influence the trajectory and definition of treatment resistance, and can revised diagnostic criteria for treatment-resistant depression improve clinical trial design and patient stratification?	How do neurophysiological biomarkers (eg, EEG gamma oscillations, entropy, scale-free dynamics) predict or differentiate early vs late responders to ketamine/esketamine infusions in TRD and can they be used to personalise dosing or administration protocols? What is the mechanistic basis for the differential effects of NMDA receptor antagonists (eg, ketamine, memantine) on proteasome activity and synaptic protein homeostasis, and how does this relate to sustained antidepressant effects beyond acute NMDA blockade? Can coadministration of dopamine modulators (eg, aripiprazole) selectively suppress ketamine-induced dissociation without compromising rapid antidepressant efficacy, and is this combination clinically superior to ketamine alone in diverse TRD populations? To what extent do ethnic, metabolic (eg, BMI) or pharmacokinetic differences (eg, route of administration: intravenous vs intranasal) influence the efficacy and safety of ketamine/esketamine in TRD, particularly in underrepresented populations such as Asians or older adults? Is nasal epithelial integrity critical for the generation of ketamine-enhanced high-frequency oscillations and downstream antidepressant effects, and could targeted intranasal delivery systems be optimised to enhance bioavailability and reduce systemic side effects?
Compassion-based therapies	What is the comparative efficacy of CFT vs other evidence-based psychotherapies (eg, CBT, ACT) for treating depression and anxiety disorders in adult populations? How do mechanisms of change (eg, self-compassion, shame reduction, emotion regulation) mediate outcomes in compassion-based interventions across different clinical and non-clinical populations? Are compassion-based therapies equally effective across diverse cultural contexts, and how do cultural variations in the conceptualisation of compassion influence treatment engagement and outcomes? What is the long-term durability of clinical benefits from compassion-based therapies beyond 12 months post-treatment, and what factors predict sustained improvement or relapse? To what extent can digital or self-guided compassion-based interventions (eg, apps, online modules) achieve comparable outcomes to therapist-delivered formats, and for which populations are they most suitable?	How can CFT and SCT be effectively adapted for autistic adults to reduce self-stigma and shame, and what are the key barriers to their feasibility and uptake in this population? What are the differential physiological and psychological mechanisms by which self-compassion interventions vs mindfulness-based interventions (eg, MSC, CMT) buffer stress reactivity in clinical populations such as GAD, and can heart rate variability or other biomarkers be used to personalise treatment selection? To what extent can brief, scalable compassion-based interventions (eg, online workshops, drop-in programmes with animal-assisted components) sustainably improve psychological well-being and reduce suicidality in high-stress student populations, and what are the optimal dosages and delivery modalities for long-term impact? How do self-compassion and compassion training interact with resilience and cognitive reappraisal to promote psychological well-being in college students, and can integrated mindfulness and self-compassion curricula (eg, MSC, CFT) be systematically embedded into university mental health support systems? Can compassion-based interventions be designed to specifically target compassion fatigue and enhance compassion satisfaction among frontline healthcare workers, and how do cultural and systemic factors influence the effectiveness of such interventions in long-term care settings?
Rumination and repetitive thinking	How do different subtypes of rumination (eg, brooding vs reflection) differentially predict longitudinal outcomes in depression, anxiety and other psychiatric disorders across diverse populations? What is the relative efficacy of cognitive-behavioural, mindfulness-based and third-wave interventions in reducing pathological repetitive thinking, and which patient characteristics moderate treatment response? To what extent do neural mechanisms underlying rumination overlap with or diverge from those of worry and other forms of repetitive negative thinking, and how do these patterns change across the lifespan? How do cultural, socioeconomic and contextual factors influence the expression, perception and clinical impact of rumination, and are current assessment tools culturally valid across global populations? Can digital phenotyping (eg, via smartphone sensors, speech patterns or social media use) reliably detect and track rumination in real time, and does real-time feedback improve intervention adherence and outcomes?	How do distinct subtypes of rumination (eg, brooding vs reflection) differentially contribute to depression phenotypes in males vs females, and can targeted interventions be designed based on these gender-specific rumination networks? To what extent does the interaction between Fear of Missing Out (FoMO) and rumination drive excessive social media use and social anxiety, and can inhibiting this interaction via digital behavioural interventions reduce psychopathology? Can RF-CBT be effectively tailored for depression subtypes such as melancholic vs anxious distress, and what biomarkers or cognitive profiles predict differential treatment response? Does reducing mind wandering through acute interventions (eg, exercise) translate into sustained improvements in cognitive functioning and mental health outcomes, and is mind wandering a transdiagnostic mechanism linking stress, rumination and learning deficits? What role do residual rumination and impaired emotional competence play in relapse vulnerability among remitted late-life depression, and can interventions targeting emotional regulation outperform traditional antidepressant maintenance in preventing recurrence?

ACT, acceptance and commitment therapy; BMI, body mass index; CBT, cognitive behavioural therapy; CFT, compassion-focused therapy; CMT, compassionate mind training; DBS, deep brain stimulation; DBT, dialectical behaviour therapy; EEG, electroencephalography; GAD, generalised anxiety disorder; MDMA, 3,4-methylenedioxymethamphetamine; MSC, mindful self-compassion; NMDA, n-methyl-D-aspartate; RF-CBT, rumination-focused cognitive behavioural therapy; SCT, self-compassion training; TMS, transcranial magnetic stimulation; TRD, treatment-resistant depression; VNS, vagus nerve stimulation.

## DISCUSSION

In this study, we presented the development and evaluation of a new automated tool to predict trendy topics in the literature for anxiety, depression and psychosis as well as to generate candidate research synthesis questions within those topics to support the prioritisation of questions for research synthesis to accelerate translation. Our approach combines topic modelling and time series forecasting to detect topics that are trending at a given timepoint and allows for the ranking of how ‘trendy’ a topic is, meaning that there is a recent surge of interest in the topic that exceeds expectations, unrelated to how popular the topic might be. We evaluated our approach using a blinded survey of experts and found that more often than not experts agreed with the model, although there was a large variability reflecting the subjective nature of the task. We used the generative capabilities of an open LLM to generate candidate research synthesis questions that fitted within each topic, introducing a novel ‘topic-augmented’ method in which additional context is provided to the model from the detected topics to guide generation, and observed that this increased the specificity of the generated questions relative to a zero-shot baseline.

The use of artificial intelligence approaches and specifically LLMs with or without augmentation to enhance and accelerate scientific research and evidence synthesis is rapidly developing. Recent years have seen a plethora of both commercial and academic approaches being developed, for example, Google’s recently released research synthesis tool that uses ‘test time diffusion’ approach to generate entire research reports based on retrieval-augmented diffusion and a self-evolutionary iterative process.^27^ While such tools may produce outputs that resemble the outputs of systematic reviews, they typically lack the key elements of being truly systematic and transparent, and as a result, there is caution that their widespread adoption may lead to a dilution of the quality of evidence.^28^ At the same time, approaches that automate individual steps in typical research synthesis workflows are advancing rapidly, including for title and abstract screening^29 30^ and data extraction.^31^ Our approach fits within the broad scope of research landscape mapping and takes inspiration from existing methods for topic modelling^25^ and trend detection;^22 23^ however, there are no previous efforts that we are aware of that develop a comprehensive approach to trend detection and research synthesis question generation in the mental health literature.

Nevertheless, our study still has several limitations. First, we currently treat all topics equivalently regardless of their size, hierarchy or whether they address mainly clinical or preclinical research. In the future, we will integrate topic classifications and other relevant classifications such as the GALENOS ontology.^15^ Second, our time series model does not fully consider temporal patterning within publication trends, such as January spikes, as the model is trained on abstracted sequences of data from 6-month periods without explicit month names. In future work, we will develop a model that is aware of the different calendar months and considers background publication trends. Another limitation is that our topic model and our time series model are built separately, leaving out the possibility of change or evolution in topics. Dynamic topic models^21^ can represent changes in topics over time; however, these are computationally expensive and complicate determination of topic identity. An additional limitation is that while some topics go through ‘wave’-like patterns of increasing and decreasing interest, these longer term patterns are not yet taken into consideration in our models. More detailed bibliometric analyses may also trace the causes of increases and decreases in interest, as in some cases, these may be traceably due to important key findings that spark or reduce subsequent interest. Our approach is currently limited to titles and abstracts; future work will explore use of full texts as well as extend our approach to automatically inform other key aspects of the choice of topics for research synthesis, namely the level of consensus in the field, and the translational or therapeutic actionability of the evidence. In particular, while our current study evaluated the specificity of generated questions, specificity alone does not imply that generated questions truly represent open research questions, as opposed to questions already addressed; this question will be evaluated in future work. Finally, we will make our approach widely available online as an interactive tool as a part of the GALENOS website.

## Clinical implications

Artificial intelligence tools promise to accelerate scientific research processes, yet many challenges remain necessitating their careful implementation and evaluation for different purposes. Our approach supports research ‘horizon scanning’: monitoring the published literature for trends and suggesting key synthesis questions to inform research prioritisation.

## Supplementary material

10.1136/bmjment-2025-302379 online supplemental file 1

## Data Availability

Data are available in a public, open access repository.
