# Supplementary material for: Automatically detecting trends and open questions from mental health publications: a Wellcome-funded GALENOS project
Source: BMJ Ment Health. 2026 Apr 2;29(1):e302379. doi: 10.1136/bmjment-2025-302379 (PMC13055325; doi:10.1136/bmjment-2025-302379)
Supplement: online supplemental file 1 [file bmjment-29-1-s001.pdf]

SUPPLEMENTARY MATERIAL

Automatically Detecting Trends and Open Questions from Mental Health Publications

Janna Hastings[1,2,3,4], Marie Wosny[2,3], Jaycee Kennett[5,6], Ava Homiar[5,7], Gin S. Malhi[5,8], Toshi A. Furukawa[9], Jennifer Potts[5], James Thomas[6] and Andrea Cipriani[5,10,11]

[1] Idiap Research Institute, Martigny, Switzerland
[2] Institute for Implementation Science in Health Care, University of Zurich
[3] School of Medicine, University of St. Gallen, Switzerland
[4] Swiss Institute of Bioinformatics, Switzerland
[5] Department of Psychiatry, University of Oxford, UK
[6] EPPI Centre, UCL Social Research Institute, University College London, UK
[7] Division of Clinical Informatics, Harvard Medical School, USA
[8] Academic Department of Psychiatry, Kolling Institute, Northern Clinical School, Faculty of Medicine and Health, The University of Sydney, NSW, Australia
[9] Kyoto University Office of Institutional Advancement and Communications, Kyoto, Japan
[10] Oxford Precision Psychiatry Lab, NIHR Oxford Health Biomedical Research Centre, Oxford, UK
[11] NIHR Oxford Health Clinical Research Facility, Oxford Health NHS Foundation Trust, Warneford Hospital, Oxford, UK

TABLE OF CONTENTS

Supplementary Figures

SUPPLEMENTARY FIGURE S1 – TOPIC DISTRIBUTION 2
SUPPLEMENTARY FIGURE S2 – TEMPORAL MENTIONS OF TOPICS AT DIFFERENT TIME SCALES 3
SUPPLEMENTARY FIGURE S3 – SENSITIVITY OF TRENDINESS PREDICTION TO PARAMETERS 4
SUPPLEMENTARY FIGURE S4 – HIERARCHY OF TOPICS IN TOPIC MODEL 4
SUPPLEMENTARY FIGURE S5 – TIME SERIES PATTERNS FOR TWO CONTESTED TOPICS 5

Supplementary Tables and other materials

SUPPLEMENTARY TABLE S1 – PUBLICATION COUNTS PER YEAR 5
SUPPLEMENTARY TABLE S2 – RESEARCH QUESTIONS GENERATED FOR 10 TOPICS 1
SUPPLEMENTARY FILE S1 – EXPERT QUESTIONNAIRE 1
ADDITIONAL SUPPLEMENTARY DATA FILES ..... **Error! Bookmark not defined.**

SUPPLEMENTARY FIGURE S1 – TOPIC DISTRIBUTION

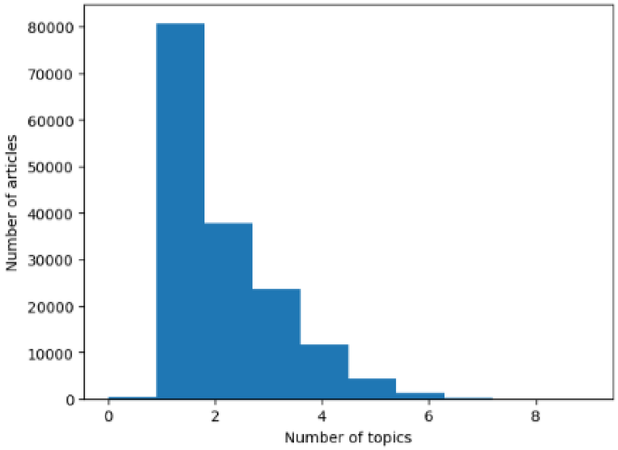

A

B

```
Efficacy of a CBT Self-Help App (Zemedy) Versus an Education, Relaxation, and Mindfulness App for IBS Goals: To test the effica
help cognitive behavioral therapy (CBT) for irritable bowel syndrome (IBS) app compared with an active control app. Background
order of gut-brain interaction that can result in significant distress, disability, and psychiatric co-morbidity. CBT is an ef
ent for IBS. Self-help CBT apps can increase accessibility but should be tested against active controls. Methods: This randomi
trial (NCT04665271) compared a CBT for IBS self-help app (Zemedy) to an active education, lifestyle management, relaxation, an
control app. A total of 453 individuals were screened in and offered allocation to treatment. Participants who actually downlo
igned app (N=267) were evenly split between the CBT app (N=136) and the active control (N=131). Follow-up data (CBT N=74, cont
collected immediately post-treatment, at which point the control group was offered crossover to CBT. Follow-up data were colle
5) and 6 (N=32) months. Primary outcomes included IBS symptom severity and IBS quality of life. Secondary outcomes included ca
visceral anxiety, fear of food, and depression. Results: At post-treatment, the CBT group improved significantly across all ou
ntrol group also improved on all outcomes except fear of food. In the intent-to-treat analysis the CBT group improved signific
n the control group on both primary and secondary outcomes except depression. Gains were maintained at 3 and 6 months, althoug
s considerable. Conclusion: Self-help CBT for IBS may be effective and can be delivered successfully through apps, although we
data on engagement. Psychoeducation about symptom management strategies, relaxation, and mindfulness are active treatment comp
T is better at addressing underlying maintaining factors like catastrophizing, visceral anxiety, and fear of food.
```

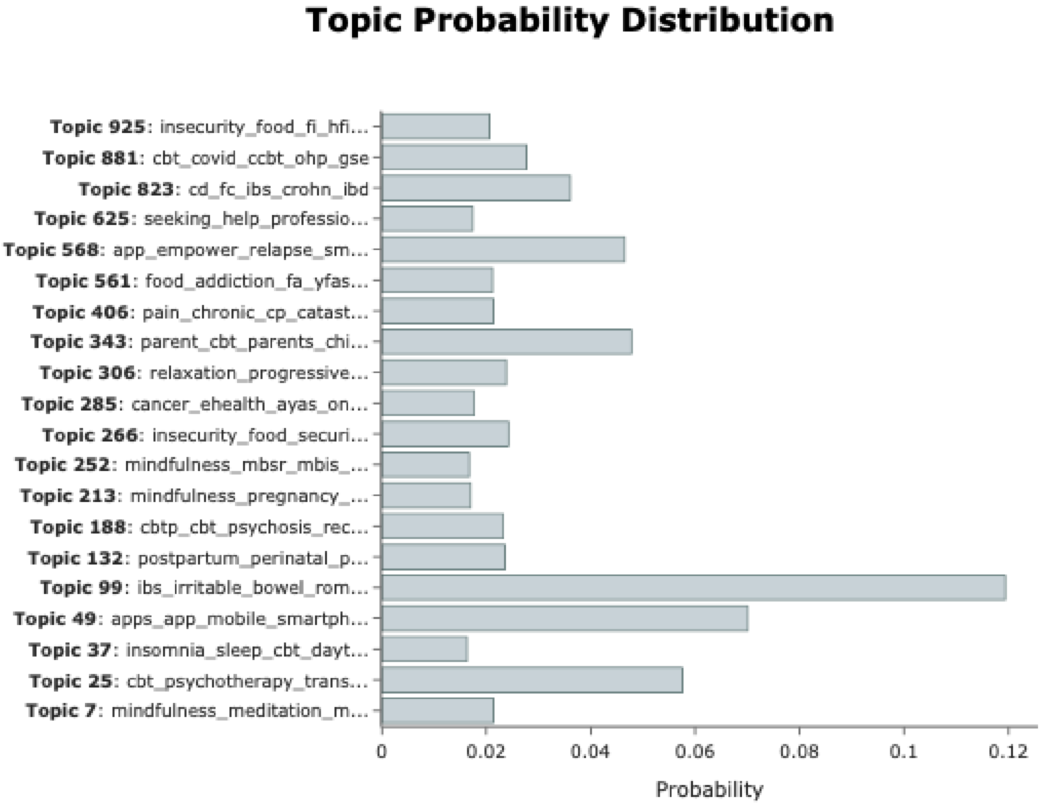

Supplementary Figure S1: (A) Histogram illustrating the distribution of topic counts per document for the probability threshold 0.08: the number of articles in the dataset is shown for each of the numbers of topics per article. (B) Example of topic probabilities assigned for one abstract.

SUPPLEMENTARY FIGURE S2 – TEMPORAL MENTIONS OF TOPICS AT DIFFERENT TIME SCALES

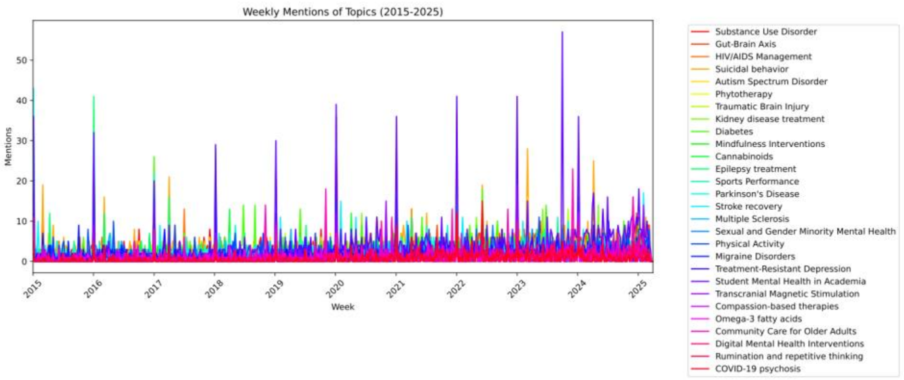

A

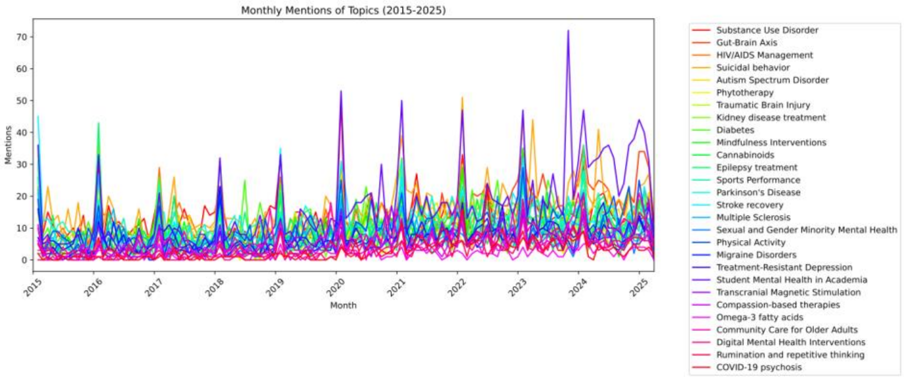

B

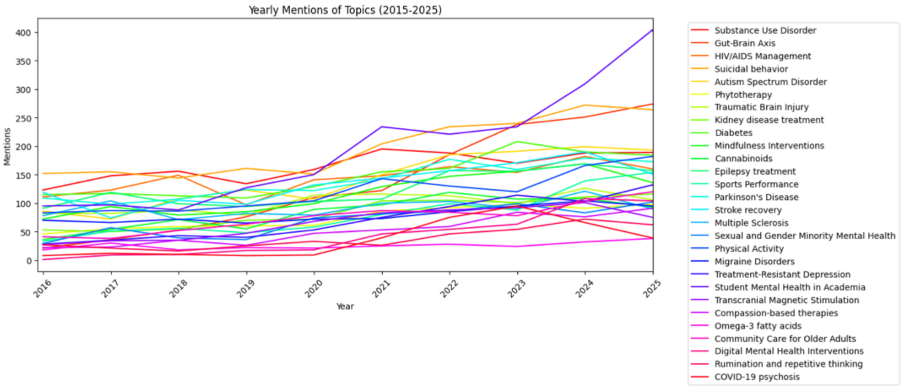

C

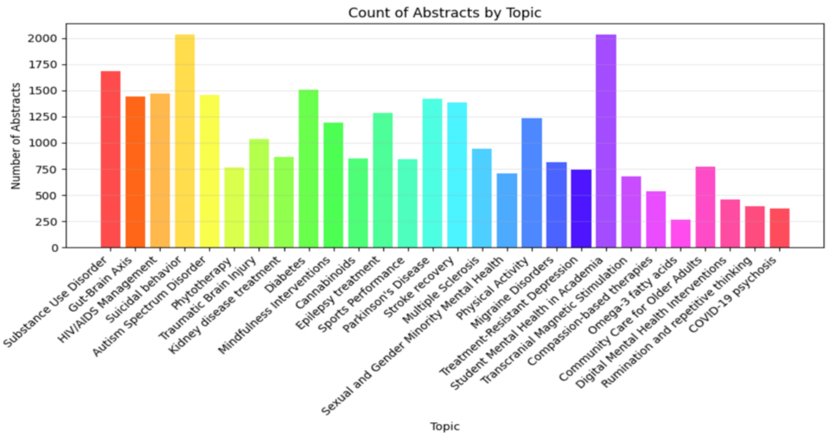

D

Supplementary Figure S2 A – weekly mentions of selected topics; B – monthly mentions of selected topics; C-yearly mentions of selected topics, showing overall trends. D – Overall sizes of the topics in terms of numbers of abstracts.

SUPPLEMENTARY FIGURE S3 – SENSITIVITY OF TRENDINESS PREDICTION TO PARAMETERS

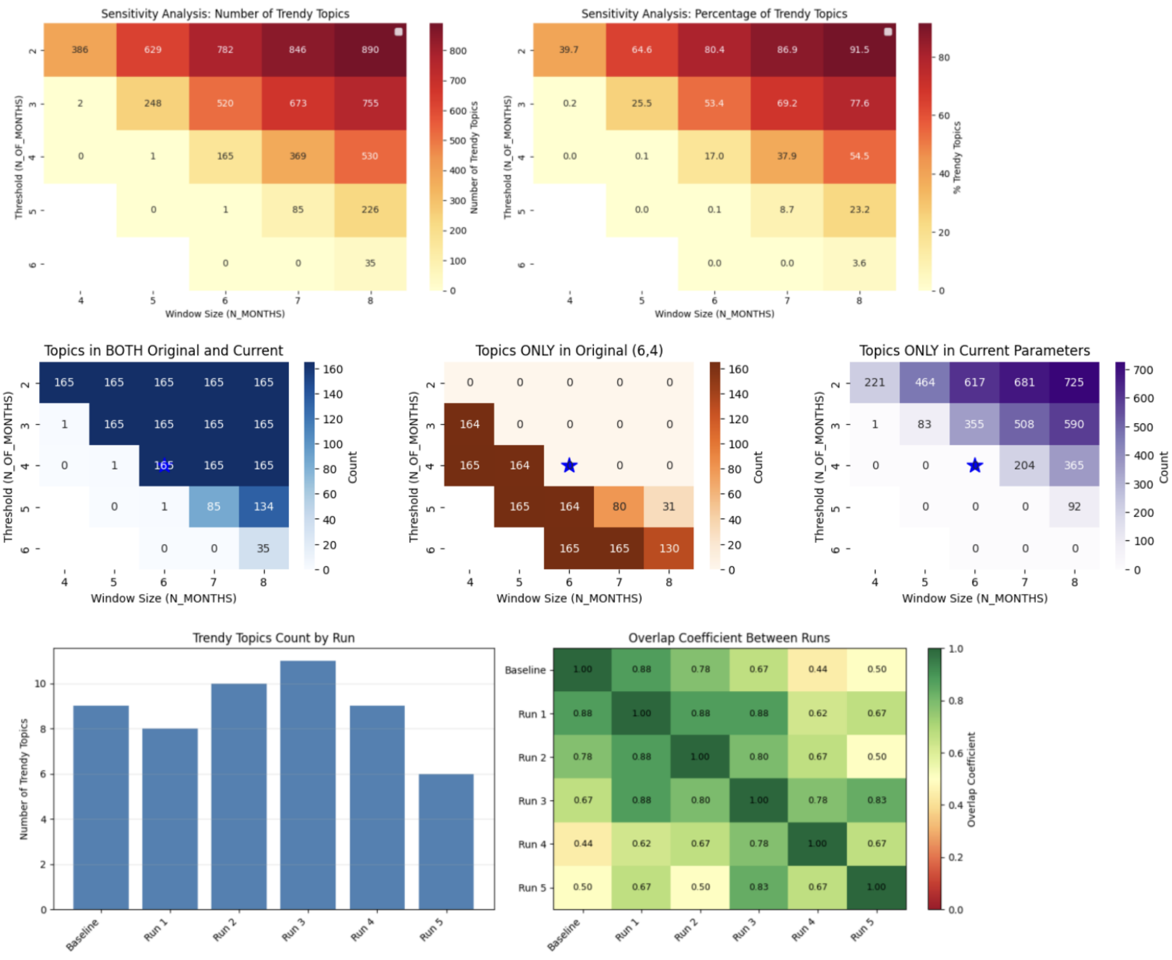

Supplementary Figure S3 (A) Sensitivity analysis of the threshold parameters 4 out of 6 months. It can be seen that the prediction of trendiness is strongly sensitive to these parameters. (B) Topic overlap in terms of identified topics under the different parameter scenarios, relationship to selected parameters. (C) Variance of trendiness prediction in top 50 topics with varying percentages of January spike data distributed across the full year.

SUPPLEMENTARY FIGURE S4 – HIERARCHY OF TOPICS IN TOPIC MODEL

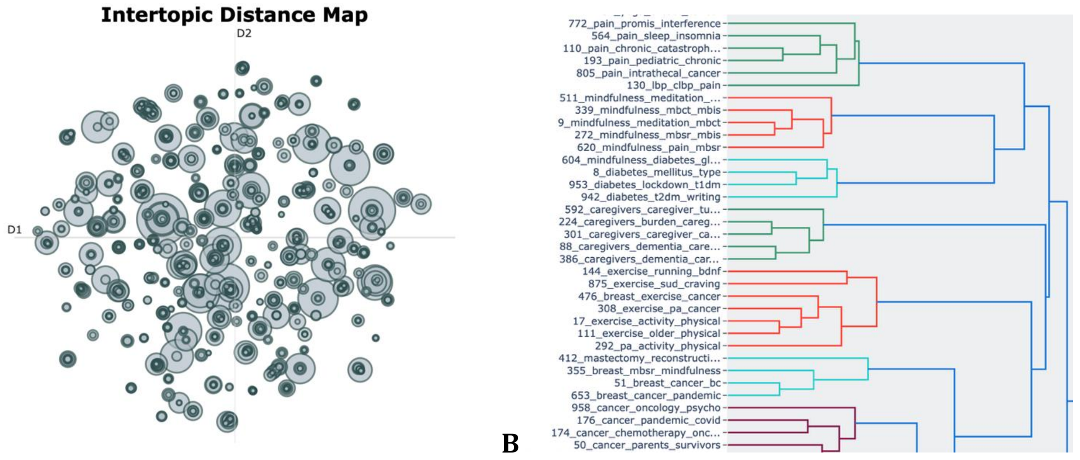

Supplementary Figure S4 (A) Topic sizes and overlaps in a two-dimensional representation of the topics, in which larger topics are shown to encompass smaller topics hierarchically. (B) An extract of the hierarchical clustering diagram for all topics.

SUPPLEMENTARY FIGURE S5 – TIME SERIES PATTERNS FOR TWO CONTESTED TOPICS

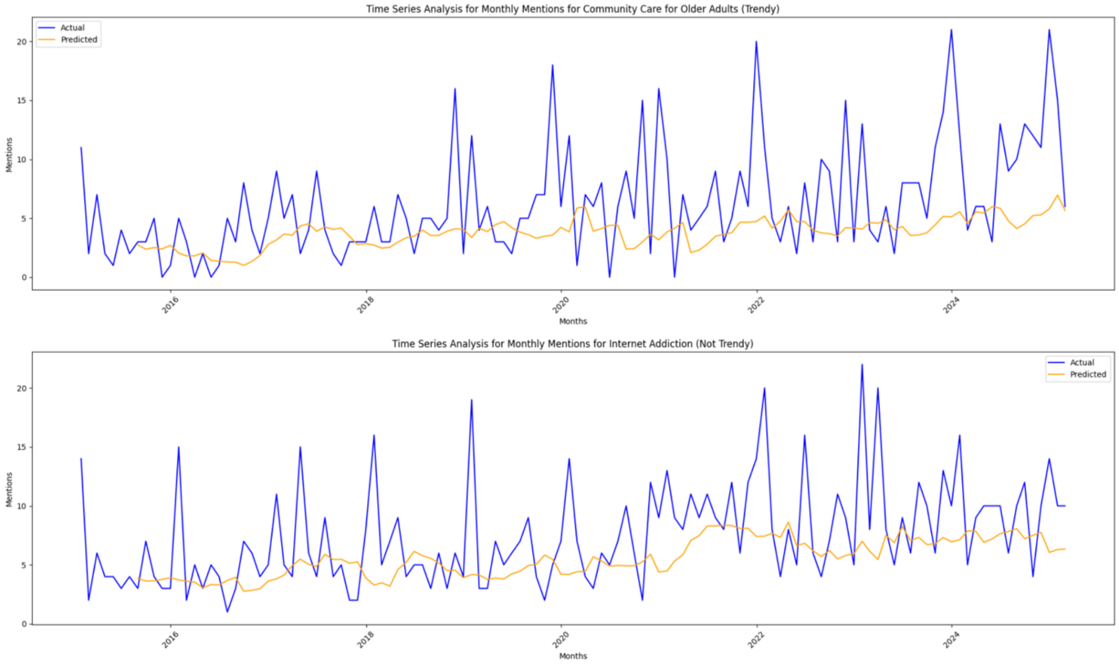

Supplementary Figure S5 shows time series patterns for topics on which experts did not agree with our model's predictions – community care for older adults, which the model ranked as trendy while the experts did not rate it as trendy, and internet addiction, which the experts rated as trendy while the model did not predict that it was trending.

SUPPLEMENTARY TABLE S1 – PUBLICATION COUNTS PER YEAR

Results for search query 'anxiety OR depression OR psychosis' in PubMed as of 15.10.2025. The average since 2000 is 7.02%.

Search query: anxiety OR depression OR psychosis		
Year	Count	Rate of Increase
2026	34	N/A
2025	50831	N/A
2024	57295	1.62832361
2023	56377	-5.7508735
2022	59817	1.35725905
2021	59016	16.6139741
2020	50608	19.5219876
2019	42342	5.30216364
2018	40210	5.21495669
2017	38217	3.79413362
2016	36820	2.91240427
2015	35778	4.96699428
2014	34085	5.25909456
2013	32382	9.50963815
2012	29570	9.25549603
2011	27065	9.18149179
2010	24789	9.29412283
2009	22681	4.55928453

2008	21692	7.13685978	
2007	20247	7.74839019	
2006	18791	13.6575334	
2005	16533	11.3033526	
2004	14854	8.24163813	
2003	13723	9.02518471	
2002	12587	3.99041639	
2001	12104	5.22472399	
2000	11503	6.50925926	
1999	10800	2.66159696	
1998	10520	4.26164519	
1997	10090	1.54991948	
1996	9936	7.28862974	
1995	9261	-1.7191977	
1994	9423	2.65824164	
1993	9179	-0.552546	
1992	9230	3.00189711	
1991	8961	-2.8196508	
1990	9221	5.02277904	
1989	8780	9.48996134	
1988	8019	1.85443922	
1987	7873	0.61341853	
1986	7825	3.51898399	
1985	7559	2.46712756	
1984	7377	3.18925724	
1983	7149	10.1540832	
1982	6490	9.36973374	
1981	5934	3.88655462	
1980	5712	2.12765957	
1979	5593	3.57407407	
1978	5400	2.97482838	
1977	5244	-6.2734584	
1976	5595	-12.413901	
1975	6388	27.5304452	
1974	5009	25.6963614	
1973	3985	-3.2297232	
1972	4118	-4.6759259	
1971	4320	0.9581678	
1970	4279	-3.4303769	
1969	4431	17.3774834	
1968	3775	53.1440162	
1967	2465	57.7095329	
1966	1563	-0.6988564	
1965	1574	-21.613546	
1964	2008	33.1564987	
1963	1508	51.7102616	

1962	994	-1.9723866	
1961	1014	7.6433121	
1960	942	0.7486631	
1959	935	12.6506024	
1958	830	0.60606061	
1957	825	-0.9603842	
1956	833	12.8726287	
1955	738	14.5962733	
1954	644	-2.8657617	
1953	663	5.07131537	
1952	631	6.22895623	
1951	594	10	
1950	540	36.3636364	
1949	396	19.2771084	
1948	332	-5.6818182	
1947	352	7.64525994	
1946	327	186.842105	
1945	114	N/A	

SUPPLEMENTARY TABLE S2 – RESEARCH QUESTIONS GENERATED FOR 10 TOPICS

Topic Name	Baseline Zero-Shot Generation Approach	Our Topic-Augmented Generation Approach
Ketamine for treatment-resistant depression	1. What is the comparative effectiveness of novel neuromodulation techniques (e.g., TMS, ketamine, esketamine, VNS, and DBS) in reducing depressive symptoms and improving functional outcomes in patients with treatment-resistant depression across different symptom profiles and comorbidities?<br>2. Among patients with treatment-resistant depression, what are the long-term (≥2 years) outcomes of pharmacological augmentation strategies (e.g., lithium, thyroid hormone, atypical antipsychotics) versus switching to alternative mechanisms of action (e.g., MDMA-assisted therapy, psilocybin), and how do these outcomes vary by biomarker profiles or genetic subtypes<br>3. How do psychosocial interventions (e.g., CBT, DBT, ACT, and behavioral activation) modify treatment response when combined with pharmacological or neuromodulatory interventions in treatment-resistant depression, and which patient subgroups benefit most from integrated models<br>4. What is the role of inflammatory, metabolic, and gut-microbiome biomarkers in predicting resistance to standard antidepressants and guiding personalized treatment selection in treatment-resistant depression, and do interventions targeting these pathways improve clinical outcomes?<br>5. How do real-world treatment sequences and adherence patterns in diverse healthcare settings influence the trajectory and definition of treatment resistance, and can revised diagnostic criteria for treatment-resistant depression improve clinical trial design and patient stratification?	1. How do neurophysiological biomarkers (e.g., EEG gamma oscillations, entropy, scale-free dynamics) predict or differentiate early vs. late responders to ketamine/esketamine infusions in treatment-resistant depression (TRD), and can they be used to personalize dosing or administration protocols?<br>2. What is the mechanistic basis for the differential effects of NMDA receptor antagonists (e.g., ketamine, memantine) on proteasome activity and synaptic protein homeostasis, and how does this relate to sustained antidepressant effects beyond acute NMDA blockade?<br>3. Can co-administration of dopamine modulators (e.g., aripiprazole) selectively suppress ketamine-induced dissociation without compromising rapid antidepressant efficacy, and is this combination clinically superior to ketamine alone in diverse TRD populations?<br>4. To what extent do ethnic, metabolic (e.g., BMI), or pharmacokinetic differences (e.g., route of administration: intravenous vs. intranasal) influence the efficacy and safety of ketamine/esketamine in TRD, particularly in underrepresented populations such as Asians or older adults?<br>5. Is nasal epithelial integrity critical for the generation of ketamine-enhanced high-frequency oscillations and downstream antidepressant effects, and could targeted intranasal delivery systems be optimized to enhance bioavailability and reduce systemic side effects?
Omega-3 fatty acids	1. What is the efficacy of omega-3 fatty acid supplementation (EPA/DHA) in preventing or delaying the onset of major depressive disorder in high-risk populations (e.g., individuals with subsyndromal symptoms or family history)?<br>2. How do different ratios of EPA to DHA in omega-3 supplements influence clinical outcomes in patients with anxiety disorders, and is there an optimal ratio for symptom reduction?<br>3. Do omega-3 fatty acids modify the trajectory of cognitive decline in older adults with mild cognitive impairment, and does effect size vary by APOE ε4 genotype?<br>4. What is the comparative effectiveness of omega-3 fatty acids versus standard pharmacological treatments (e.g., SSRIs) for treating moderate-to-severe depression in randomized controlled trials with long-term follow-up (>6 months)?<br>5. In children and adolescents with ADHD, does omega-3 supplementation improve core symptoms (inattention, hyperactivity) and executive function, and are outcomes moderated by baseline omega-3 status or genetic polymorphisms?	1. What is the optimal EPA-to-DHA ratio in omega-3 supplementation for maximizing antidepressant effects in major depressive disorder, and how do individual differences in inflammatory status, genetics, and baseline n-3 PUFA levels modulate treatment response?<br>2. To what extent do deficits in erythrocyte membrane polyunsaturated fatty acids (PUFAs) reflect or contribute to neurobiological dysfunction in adolescent depression, and can targeted omega-3 supplementation restore membrane fluidity and mitigate symptom severity?<br>3. Can dietary omega-3 fatty acids (particularly ALA from sources like walnuts) exert meaningful neuroprotective and anxiolytic effects via the gut-brain axis, and how does their inefficient conversion to EPA and DHA impact clinical efficacy compared to direct supplementation with fish-derived EPA/DHA?<br>4. Do genetically predicted levels of omega-3 fatty acids causally influence the risk of specific mental disorders (e.g., OCD, bipolar disorder, schizophrenia), and can Mendelian randomization findings be translated into personalized prevention strategies based on polyunsaturated fatty acid metabolism profiles?<br>5. How do omega-3 fatty acids interact with other nutritional interventions (e.g., curcumin, magnesium, probiotics) in craniofacial inflammatory conditions to produce synergistic anxiolytic effects, and what standardized outcome measures are needed to enable cross-study comparison and clinical translation?

Community Care for Older Adults	1. What are the most effective integrated care models for improving mental health outcomes in older adults living in community settings, and how do these models vary by socioeconomic or geographic context?<br>2. How does the implementation of technology-assisted care (e.g., telehealth, remote monitoring) impact social isolation, medication adherence, and hospital utilization among older adults with chronic conditions in underserved communities?<br>3. What are the long-term effects of formal and informal caregiver support programs on the health, well-being, and institutionalization rates of older adults, and which program components are most predictive of sustained outcomes?<br>4. To what extent do culturally tailored community care interventions reduce disparities in access and outcomes for older adults from racial, ethnic, or linguistic minority groups?<br>5. How do policy-level factors (e.g., funding mechanisms, workforce incentives, interagency coordination) influence the scalability and sustainability of community-based care programs for older adults across different healthcare systems?	1. How can collaborative, community-based interventions involving Community Health Workers (CHWs) and care home staff be systematically optimized to improve the identification, assessment, and management of depression among older adult residents in long-term care homes, particularly those with dementia?<br>2. What specific types of home- and community-based activation interventions (e.g., behavioral activation, social engagement, outdoor activity) are most effective in reducing depression and improving psychological well-being among homebound older adults with noncommunicable diseases, and how do these effects vary by sociodemographic or functional status?<br>3. To what extent can smart home technologies (SHTs) with AI-driven monitoring and personalized alerts be designed and implemented in a user-centered, accessible, and ethically sound manner to enable early detection and just-in-time intervention for mental health decline in older adults aging in place?<br>4. How do collaborative models integrating social prescribing, primary care, and care home activity coordinators impact long-term mental health outcomes and quality of life for older adult residents, and what are the key barriers to sustaining these partnerships in diverse community settings?<br>5. Can tailored, technology-enhanced interventions (e.g., smartphone-based cognitive games, robot-assisted gait training) be effectively scaled and adapted for homebound or low-resource older adult populations to simultaneously address cognitive decline, mobility, depression, and social isolation as interconnected domains of care?
Compassion-based therapies	1. What is the comparative efficacy of compassion-focused therapy (CFT) versus other evidence-based psychotherapies (e.g., CBT, ACT) for treating depression and anxiety disorders in adult populations?<br>2. How do mechanisms of change (e.g., self-compassion, shame reduction, emotion regulation) mediate outcomes in compassion-based interventions across different clinical and non-clinical populations?<br>3. Are compassion-based therapies equally effective across diverse cultural contexts, and how do cultural variations in the conceptualization of compassion influence treatment engagement and outcomes?<br>4. What is the long-term durability of clinical benefits from compassion-based therapies beyond 12 months post-treatment, and what factors predict sustained improvement or relapse?<br>5. To what extent can digital or self-guided compassion-based interventions (e.g., apps, online modules) achieve comparable outcomes to therapist-delivered formats, and for which populations are they most suitable?	1. How can compassion-focused therapies (CFT) and self-compassion training (SCT) be effectively adapted for autistic adults to reduce self-stigma and shame, and what are the key barriers to their feasibility and uptake in this population?<br>2. What are the differential physiological and psychological mechanisms by which self-compassion interventions versus mindfulness-based interventions (e.g., MSC, CMT) buffer stress reactivity in clinical populations such as generalized anxiety disorder (GAD), and can heart rate variability or other biomarkers be used to personalize treatment selection?<br>3. To what extent can brief, scalable compassion-based interventions (e.g., online workshops, drop-in programs with animal-assisted components) sustainably improve psychological wellbeing and reduce suicidality in high-stress student populations, and what are the optimal dosages and delivery modalities for long-term impact?<br>4. How do self-compassion and compassion training interact with resilience and cognitive reappraisal to promote psychological wellbeing in college students, and can integrated mindfulness and self-compassion curricula (e.g., MSc, CFT) be systematically embedded into university mental health support systems?<br>5. Can compassion-based interventions be designed to specifically target compassion fatigue and enhance compassion satisfaction among frontline healthcare workers, and how do cultural and systemic factors influence the effectiveness of such interventions in long-term care settings?
Student Mental Health in Academia	1. What are the most effective scalable interventions for reducing anxiety and depression among university students across diverse cultural and institutional contexts?<br>2. How do academic structures (e.g., grading systems, workload expectations, tenure-track pressures on faculty) contribute to student mental health outcomes, and which structural reforms show the strongest evidence of improvement?<br>3. What is the long-term impact of campus-based mental health services on academic retention, graduation rates, and post-graduation mental health outcomes among students with diagnosed conditions?	1. How do longitudinal changes in emotional intelligence and coping strategies among medical and undergraduate students influence the prevalence of anxiety, depression, and stress across academic years, particularly during high-pressure transitions (e.g., preclinical to clinical, or early to late PhD)?<br>2. What institutional and systemic interventions—such as flexible academic policies, integrated mental health services, or industry-academia partnerships—are most effective in mitigating work-study conflict and its cascading effects on mental health and academic performance among undergraduate and graduate students in high-demand disciplines like medicine and built environment?<br>3. To what extent do gender, cultural context, and geographic location (e.g., urban vs. non-metropolitan universities) moderate the relationship between academic stress, social support, and mental health outcomes in student populations, particularly in under-

	4. How do digital mental health tools (e.g., apps, teletherapy, AI chatbots) compare to traditional in-person services in terms of accessibility, engagement, and efficacy for marginalized student populations (e.g., first-generation, LGBTQ+, international students)?<br>5. What role does stigma within academic cultures play in deterring students from seeking mental health support, and which campus-level anti-stigma interventions have demonstrated measurable impact?	researched regions such as Bangladesh or rural Indonesia?<br>4. Can non-pharmacological, activity-based interventions (e.g., basketball training, VR-based simulations, or stress inoculation training) be systematically scaled and adapted across diverse university settings to reliably reduce psychological distress and improve academic self-efficacy in both undergraduate and graduate students?<br>5. How can university counselors and mental health support systems be optimized—through training, technology (e.g., digital health records, peer networks), and humanistic care models—to effectively identify, intervene, and support students with severe mental illnesses (e.g., schizophrenia) without exacerbating stigma or social isolation?
Rumination and repetitive thinking	1. How do different subtypes of rumination (e.g., brooding vs. reflection) differentially predict longitudinal outcomes in depression, anxiety, and other psychiatric disorders across diverse populations?<br>2. What is the relative efficacy of cognitive-behavioral, mindfulness-based, and third-wave interventions in reducing pathological repetitive thinking, and which patient characteristics moderate treatment response?<br>3. To what extent do neural mechanisms underlying rumination overlap with or diverge from those of worry and other forms of repetitive negative thinking, and how do these patterns change across the lifespan?<br>4. How do cultural, socioeconomic, and contextual factors influence the expression, perception, and clinical impact of rumination, and are current assessment tools culturally valid across global populations?<br>5. Can digital phenotyping (e.g., via smartphone sensors, speech patterns, or social media use) reliably detect and track rumination in real time, and does real-time feedback improve intervention adherence and outcomes?	1. How do distinct subtypes of rumination (e.g., brooding vs. reflection) differentially contribute to depression phenotypes in males versus females, and can targeted interventions be designed based on these gender-specific rumination networks?<br>2. To what extent does the interaction between Fear of Missing Out (FoMO) and rumination drive excessive social media use and social anxiety, and can inhibiting this interaction via digital behavioral interventions reduce psychopathology?<br>3. Can rumination-focused cognitive behavioral therapy (RF-CBT) be effectively tailored for depression subtypes such as melancholic versus anxious distress, and what biomarkers or cognitive profiles predict differential treatment response?<br>4. Does reducing mind wandering through acute interventions (e.g., exercise) translate into sustained improvements in cognitive functioning and mental health outcomes, and is mind wandering a transdiagnostic mechanism linking stress, rumination, and learning deficits?<br>5. What role do residual rumination and impaired emotional competence play in relapse vulnerability among remitted late-life depression, and can interventions targeting emotional regulation outperform traditional antidepressant maintenance in preventing recurrence?
Gut-Brain Axis	1. What is the efficacy and consistency of probiotic interventions in improving symptoms of anxiety and depression across randomized controlled trials, and how do strain-specific effects modify outcomes?<br>2. How do gut microbiota composition and diversity differ in individuals with autism spectrum disorder (ASD) compared to neurotypical controls, and are these differences consistent across geographic and demographic populations?<br>3. What is the role of the vagus nerve in mediating gut-brain signaling in humans with psychiatric or neurological disorders, and how do vagal tone interventions (e.g., vagus nerve stimulation) impact clinical outcomes?<br>4. Do dietary patterns (e.g., Mediterranean, high-fiber, ketogenic) that modulate the gut microbiome lead to measurable changes in brain structure, function, or behavior in longitudinal human studies?<br>5. To what extent do antibiotics and proton pump inhibitors (PPIs) alter the gut-brain axis and increase the risk of developing mood or cognitive disorders over time, and are these effects dose- or duration-dependent?	1. How do specific microbial metabolites (e.g., SCFAs, bile acids, tryptophan derivatives) mechanistically mediate the bidirectional signaling along the gut-brain axis to influence distinct psychiatric symptoms such as anxiety, depression, and cognitive decline across different disease contexts (e.g., IBD, T1D, long COVID, cholestasis)?<br>2. Can fecal microbiota transplantation (FMT) or targeted probiotic interventions reliably reverse dysbiosis-induced neuropsychiatric phenotypes, and what are the critical bacterial taxa and metabolic pathways that must be restored for therapeutic efficacy in humans?<br>3. To what extent do host genetic, dietary, and environmental factors (e.g., capsaicin, zinc exposure) modulate the composition and function of the gut microbiota to exacerbate or ameliorate mental health outcomes, and how can these interactions be standardized for personalized microbiome-based therapies?<br>4. What are the causal versus correlative relationships between gut microbiota composition changes and psychopathological symptoms in anorexia nervosa and other eating disorders, and can microbial signatures serve as validated biomarkers for diagnosis or treatment monitoring?<br>5. How do interventions such as Sirt6 activators (e.g., icariside II) or hydrogen-rich water exert their neurobehavioral effects via the gut-brain axis—through direct microbial modulation, barrier restoration, or metabolite regulation—and can these mechanisms be translated into clinically viable treatments for depression, Alzheimer's, or post-stroke conditions?
Transcranial Magnetic Stimulation	1. What is the comparative efficacy and durability of response for different TMS protocols (e.g., theta-burst vs. 10 Hz rTMS) in treating treatment-resistant depression across diverse patient populations?	1. What are the optimal neurophysiological biomarkers (e.g., qEEG patterns, white matter micromorphology, frontal theta cordance) that can reliably predict remission or response to rTMS, iTBS, or dTMS in treatment-resistant depression, and how can these be validated

	2. How do individual neuroanatomical and neurophysiological biomarkers (e.g., functional connectivity, cortical excitability) predict response to TMS in major depressive disorder and other psychiatric conditions?<br>3. What is the long-term safety and cognitive impact of repeated TMS courses over multiple years in patients with chronic psychiatric or neurological disorders?<br>4. Can TMS be effectively and safely delivered in real-world, non-research clinical settings (e.g., primary care) with equivalent outcomes to those observed in controlled trials?<br>5. What is the role of TMS in treating non-depressive psychiatric conditions (e.g., PTSD, OCD, schizophrenia) when used as an adjunct to standard therapies, and which patient subgroups benefit most?	across independent, large-scale datasets?<br>2. How do different TMS coil types (F8, H1) and stimulation protocols (rTMS, iTBS, piTBS*2, aiTBS) differentially modulate circuit-level dynamics in the DLPFC and midline structures (e.g., cingulate, precuneus), and what are the cell-type-specific mechanisms (e.g., IT vs. PT neurons) underlying their antidepressant effects?<br>3. Can sustained remission in treatment-resistant depression be achieved through maintenance TMS protocols, and if so, what are the most effective dosing schedules (pulses, frequency, burst patterns) and target regions (DLPFC, midline) for long-term efficacy without cognitive decline?<br>4. What is the mechanistic basis for the differential effects of sham vs. active TMS in clinical trials, and how do placebo responses interact with neuroimaging (fMRI, PET) and electrophysiological (qEEG) markers to confound or clarify treatment outcomes?<br>5. To what extent do baseline white matter microstructure (e.g., NDI, FWF) and functional network dynamics (e.g., DMN, SN, CEN energy landscapes) predict individual response trajectories to TMS, and can these be leveraged to personalize coil placement, pulse parameters, or combination therapies (e.g., rTMS + tDCS)?
COVID-19 psychosis	1. What is the incidence and prevalence of new-onset psychosis in individuals with and without pre-existing psychiatric conditions following SARS-CoV-2 infection, and how does it vary by age, sex, and geographic region?<br>2. What are the relative contributions of direct neuroinflammatory mechanisms, hypoxic injury, cytokine storms, and psychosocial stressors (e.g., isolation, grief, economic hardship) to the development of COVID-19-associated psychosis?<br>3. How do clinical features, treatment response, and long-term outcomes of COVID-19-related psychosis differ from those of idiopathic psychotic disorders such as schizophrenia or bipolar disorder with psychotic features?<br>4. Are there specific biomarkers (e.g., inflammatory cytokines, autoantibodies, neuroimaging signatures) that can reliably distinguish COVID-19 psychosis from other etiologies of psychosis, and can they predict prognosis or treatment response?<br>5. What are the most effective pharmacological and psychosocial interventions for managing acute and persistent psychosis in the context of active or recent SARS-CoV-2 infection, and how should management be adapted in patients with comorbid medical complications?	1. To what extent does SARS-CoV-2 infection directly trigger acute psychotic symptoms (e.g., delusions, hallucinations) via neuroinflammatory, neurovascular, or neurotransmitter pathways, versus acting as a stressor that unmaskes underlying psychiatric vulnerability?<br>2. Are there distinct clinical, neuroimaging, or biomarker profiles differentiating COVID-19-associated psychosis from idiopathic schizophrenia or psychosis triggered by other viral infections (e.g., influenza, HSV), and can these inform targeted treatment approaches?<br>3. What is the long-term psychiatric prognosis for individuals who develop acute psychosis during or shortly after COVID-19 infection — does it typically resolve, or does it confer a higher risk of chronic psychotic disorders such as schizophrenia or schizoaffective disorder?<br>4. How do factors such as reinfection, severity of acute respiratory illness, hospitalization, and pre-existing psychosocial stressors interact to modulate the risk and presentation of neuropsychiatric sequelae, including psychosis, in the post-acute and long-COVID phases?<br>5. What are the underlying neurobiological mechanisms (e.g., fNIRS-identified DLPFC connectivity changes, cytokine-driven neurotoxicity, or autoimmunity) that mediate the transition from acute respiratory coronavirus infection to psychotic symptoms, and can these be targeted for early intervention?
Digital Mental Health Interventions	1. What is the comparative effectiveness of different types of digital mental health interventions (e.g., CBT apps, chatbots, virtual reality, AI-driven platforms) across diverse populations (e.g., adolescents, older adults, low-income communities) for common mental health conditions such as depression and anxiety?<br>2. How do long-term engagement and adherence patterns in digital mental health interventions predict clinical outcomes, and what digital design features (e.g., gamification, personalization, reminders) most strongly sustain usage beyond 3 months?<br>3. To what extent do digital mental health interventions reduce disparities in access to care for underserved populations, and what implementation barriers (e.g., digital literacy, infrastructure, cultural relevance) moderate their equity impact?<br>4. What is the clinical and ethical safety profile of AI-driven digital mental health tools, particularly regarding bias in algorithmic	1. How can digital mental health interventions (DMHIs) be optimized to sustain long-term engagement among adolescents and young people, particularly in marginalized or neurodivergent populations where current studies show low representation and moderate retention rates?<br>2. What are the ethical and privacy implications of third-party tracking technologies on mental health websites, and how can user data be protected without deterring help-seeking behavior among teens and youth using digital platforms?<br>3. For young people with severe mental health symptoms, why does increased use of DMHIs not consistently correlate with clinical improvement, and what unmeasured moderating factors (e.g., offline support, socioeconomic context) influence this relationship?<br>4. How can digital interventions be tailored to address a broader spectrum of mental health conditions beyond anxiety and depression in adolescents and youth, particularly for underrepresented populations such as LGBTQ+ individuals and those from socioeconomically deprived backgrounds?<br>5. What design and delivery features (e.g., session duration, frequency, modality, human

	diagnosis, data privacy, and inappropriate escalation of risk in vulnerable users?<br>5. How effective are hybrid models (digital + human support) compared to fully digital or fully in-person interventions for treating moderate-to-severe mental health conditions, and which components of human support (e.g., coaching, therapy, monitoring) add the most value?	support) maximize the effectiveness of digital mental health interventions for specific adolescent and young adult subgroups, and how can closed-loop, AI-driven personalization be safely integrated into real-world clinical workflows?

SUPPLEMENTARY FILE S1 – EXPERT QUESTIONNAIRE

GALENOS: Understanding Topic Trends in the Mental Health Literature

We would like to better understand the development of interest (publication counts) in research topics and themes across the published literature in the domain of early interventions and mechanistic studies in anxiety, depression and psychosis.

In order to do this, we have trained an AI-based model to detect trendy topics, defined as topics that have shown a substantial increase in interest in the recent six months relative to earlier levels of interest. We distinguish trendy topics from popular topics, which are those topics where a high level of interest has been sustained over time but is not specifically increasing recently.

We need your help! In order to help us evaluate our AI-based model, we have designed the following task for experts from the field to answer. We have randomly selected some of the topics that are predicted as trendy and interspersed them with randomly selected topics that are detected as popular but not trendy as well as those that are neither predicted as trendy nor as popular. Your task is to decide based on the description of a topic and your understanding of the research literature which category you think that topic falls into. Please note that this should be based on your existing knowledge of the overall field and literature, not your opinion of the value of the topic. Please do not explicitly base the response on a search of the topic but rather your own knowledge.

Ideally, for each question, you can choose one of the three options. However, if you cannot for a given question, please select 'other' and give an explanation. At the end, you have an option to give us open feedback and suggestions for the project as a whole.

Thank you very much for your valuable time and contribution to the GALENOS project!

Topic: Music therapy

Research in this topic area is characterised by words such as ''music, listening, musical, therapy, mt, singing, sound, emotions, healing, receptive, relaxation, students, sessions, psychology, gim". An exemplary recent publication in this topic area is:
Gómez-Restrepo et al. *Outcomes and experiences of music workshops for adolescents with depression and anxiety: An exploratory noncontrolled trial in Bogotá*
https://pubmed.ncbi.nlm.nih.gov/39623481/

- ◯ Trending recently
- ◯ Popular but not specifically trending recently
- ◯ Neither popular nor trending recently
- ◯ Other...

Topic: Ketamine for treatment-resistant depression

Research in this topic area is characterised by words such as ''ketamine, esketamine, antidepressant, resistant, trd, infusion, rapid, nmda, infusions, methyl, intravenous, intranasal, administration, antagonist, aspartate". An exemplary recent publication in this topic area is:
Ohtani et al. *Efficacy and safety of intravenous ketamine treatment in Japanese patients with treatment-resistant depression: A double-blind, randomized, placebo-controlled trial*
https://pubmed.ncbi.nlm.nih.gov/39210712/

- Trending recently
- Popular but not specifically trending recently
- Neither popular nor trending recently
- Other...

Topic: Omega-3 fatty acids
Research in this topic area is characterised by words such as "omega, fatty, acids, pufas, dha, polyunsaturated, epa, pufa, acid, fish, docosahexaenoic, supplementation, eicosapentaenoic, fa, oil". An exemplary recent publication in this topic area is:
Mongan et al. *Longitudinal Trajectories of Plasma Polyunsaturated Fatty Acids and Associations With Psychosis Spectrum Outcomes in Early Adulthood*
https://pubmed.ncbi.nlm.nih.gov/38631425/

- Trending recently
- Popular but not specifically trending recently
- Neither popular nor trending recently
- Other...

Topic: Community Care for Older Adults
Research in this topic area is characterised by words such as "older, adults, residents, ba, care, homes, collaborative, community, pearls, home, chws, homebound, activation, intervention, services". An exemplary recent publication in this topic area is:
Feng et al. *Latent profile analysis of depression in older adults spouse caregivers*
https://pmc.ncbi.nlm.nih.gov/articles/PMC11932916/

- Trending recently
- Popular but not specifically trending recently
- Neither popular nor trending recently
- Other...

Topic: Compassion-based therapies
Research in this topic area is characterised by words such as "compassion, cft, compassionate, self, criticism, cmt, focused, mindfulness, shame, msc, scs, kindness, training, students, sc". An exemplary recent publication in this topic area is:
Riebel et al. *Self-compassion as an antidote to self-stigma and shame in autistic adults*
https://pubmed.ncbi.nlm.nih.gov/39959965/

- Trending recently
- Popular but not specifically trending recently
- Neither popular nor trending recently
- Other...

Topic: Grief and Bereavement
Research in this topic area is characterised by words such as "grief, bereavement, bereaved, pgd, prolonged, complicated, loss, death, grieving, loved, deceased, lost, mourning, pg, posttraumatic". An exemplary recent
publication in this topic area is:
Sandler et al. *Trajectories of children's intrusive grief and association with baseline family and child factors and long-term outcomes in young adulthood*

https://pubmed.ncbi.nlm.nih.gov/39703004/

- Trending recently
- Popular but not specifically trending recently
- Neither popular nor trending recently
- Other...

Topic: Student Mental Health in Academia
Research in this topic area is characterised by words such as ''students, academic, university, medical, undergraduate, college, student, career, stress, prevalence, phd, universities, year, coping, graduate". An exemplary recent publication in this topic area is:
Li et al. *Associations between overprotective parenting style and academic anxiety among Chinese high school students*
https://pubmed.ncbi.nlm.nih.gov/40069842/

- Trending recently
- Popular but not specifically trending recently
- Neither popular nor trending recently
- Other...

Topic: Rumination and repetitive thinking
Research in this topic area is characterised by words such as ''rumination, rnt, wandering, thinking, rfcbt, repetitive, ruminative, worry, mind, brooding, mw, negative, ptq, thoughts, perseverative". An exemplary recent publication in this topic area is:
Wang et al. *Negative rumination in depression subtypes with melancholic features and anxious distress*
https://pmc.ncbi.nlm.nih.gov/articles/PMC11865946/

- Trending recently
- Popular but not specifically trending recently
- Neither popular nor trending recently
- Other...

Topic: Gut-Brain Axis
Research in this topic area is characterised by words such as ''gut, microbiota, microbiome, probiotics, axis, intestinal, microbial, probiotic, dysbiosis, bacteria, composition, fecal, metabolites, bacterial, mice". An exemplary recent publication in this topic area is:
Li et al. *Unraveling the Metabolic and Microbiome Signatures in Fecal Samples of Pregnant Women with Prenatal Depression*
https://pubmed.ncbi.nlm.nih.gov/40137144/

- Trending recently
- Popular but not specifically trending recently
- Neither popular nor trending recently
- Other...

Topic: Women's reproductive health issues

Research in this topic area is characterised by words such as ''endometriosis, pelvic, dyspareunia, pain, women, dysmenorrhea, endometrial, uterine, infertility, adenomyosis, ovarian, gynecological, cysts, reproductive, uterus". An exemplary recent publication in this topic area is:
Panvino et al. *Endometriosis in Adolescence: A Narrative Review of the Psychological and Clinical Implications*
https://pubmed.ncbi.nlm.nih.gov/40075795/

- ○ Trending recently
- ○ Popular but not specifically trending recently
- ○ Neither popular nor trending recently
- ○ Other...

Topic: Mental health stigma
Research in this topic area is characterised by words such as ''stigma, internalized, attitudes, stigmatizing, illness, stigmatization, ismi, seeking, self, discrimination, schizophrenia, vignette, esteem, perceived, internalised". An exemplary recent publication in this topic area is:
Albuquerque et al. *Decoding Prejudice: Understanding Patterns of Adolescent Mental Health Stigma*
https://pubmed.ncbi.nlm.nih.gov/40004924/

- ○ Trending recently
- ○ Popular but not specifically trending recently
- ○ Neither popular nor trending recently
- ○ Other...

Topic: Social Isolation
Research in this topic area is characterised by words such as ''loneliness, lonely, isolation, older, social, ucla, adults, connectedness, basil, pandemic, intergenerational, people, covid, feeling, 19". An exemplary recent publication in this topic area is:
Cho et al. *Loneliness and depression: the mediating effect of perceived social strain in older adults with diabetes-a cross-sectional study*
https://pubmed.ncbi.nlm.nih.gov/39930886/

- ○ Trending recently
- ○ Popular but not specifically trending recently
- ○ Neither popular nor trending recently
- ○ Other...

Topic: Neuroplasticity
Research in this topic area is characterised by words such as ''synaptic, plasticity, synapses, ltp, potentiation, neurons, calcium, synapse, presynaptic, dependent, postsynaptic, ampar, term, excitatory, ca1". An exemplary recent publication in this topic area is:
Sened et al. *Inter-brain plasticity as a mechanism of change in psychotherapy: A proof of concept focusing on test anxiety*
https://pubmed.ncbi.nlm.nih.gov/39832304/

- ○ Trending recently
- ○ Popular but not specifically trending recently
- ○ Neither popular nor trending recently
- ○ Other...

Topic: Internet Addiction
Research in this topic area is characterised by words such as "internet, addiction, ia, piu, problematic, iat, iad, students, adolescents, addictive, use, addicted, excessive, college, addictions". An exemplary recent
publication in this topic area is:
Long et al. *The associations between PTSD symptom clusters, insomnia, and depressive symptoms in Chinese adolescents with low and high levels of internet addiction: A cross-lagged network analysis*
https://pubmed.ncbi.nlm.nih.gov/40048205/

- ◯ Trending recently
- ◯ Popular but not specifically trending recently
- ◯ Neither popular nor trending recently
- ◯ Other...

Topic: Eye Movement Desensitization and Reprocessing (EMDR) Therapy
Research in this topic area is characterised by words such as "emdr, reprocessing, desensitization, eye, movement, ptsd, traumatic, trauma, desensitisation, posttraumatic, protocol, memories, therapy, event, randomized". An exemplary recent
publication in this topic area is:
Thunnissen et al. *Negative Flashforward Imagery in Adolescent Social Anxiety Disorder: A Pilot Study of Imagery Reports and a Short EMDR Intervention*
https://pubmed.ncbi.nlm.nih.gov/39363533/

- ◯ Trending recently
- ◯ Popular but not specifically trending recently
- ◯ Neither popular nor trending recently
- ◯ Other...

Topic: Seasonal Affective Disorder
Research in this topic area is characterised by words such as "light, seasonal, lighting, blt, blue, bright, sad, circadian, winter, lux, seasonality, daylight, morning, wavelength, glasses". An exemplary recent
publication in this topic area is:
Chen et al. *Increased functional connectivity between the midbrain and frontal cortex following bright light therapy in subthreshold depression: A randomized clinical trial*
https://pubmed.ncbi.nlm.nih.gov/37971845/

- ◯ Trending recently
- ◯ Popular but not specifically trending recently
- ◯ Neither popular nor trending recently
- ◯ Other...

Topic: Mental Imagery Rescripting Therapy
Research in this topic area is characterised by words such as "imagery, rescripting, images, vividness, synchrony, intrusive, memories, positive, anhedonia, ir, referent, negative, cbm, ri, affect". An exemplary recent
publication in this topic area is:
Sivarajah et al. *Brief imagery based metacognitive intervention for flashforwards in psychosis: a fixed baseline case series*
https://pubmed.ncbi.nlm.nih.gov/39623761/

- Trending recently
- Popular but not specifically trending recently
- Neither popular nor trending recently
- Other...

Topic: Transcranial Magnetic Stimulation
Research in this topic area is characterised by words such as "rtms, tms, transcranial, stimulation, magnetic, repetitive, coil, resistant, itbs, dtms, pulses, sham, burst, dlpfc, remission". An exemplary recent publication in this topic area is:
Su et al. *Unveiling the dynamic effects of major depressive disorder and its rTMS interventions through energy landscape analysis*
https://pubmed.ncbi.nlm.nih.gov/40109660/

- Trending recently
- Popular but not specifically trending recently
- Neither popular nor trending recently
- Other...

Topic: COVID-19 psychosis
Research in this topic area is characterised by words such as "infection, covid, 19, psychosis, psychotic, cov, sars, coronavirus, case, delusions, acute, pneumonia, hallucinations, neuropsychiatric, respiratory". An exemplary recent publication in this topic area is:
Yang et al. *Acute onset psychiatric diseases after SARS-CoV-2 virus infection among pediatric patients*
https://pubmed.ncbi.nlm.nih.gov/39445192/

- Trending recently
- Popular but not specifically trending recently
- Neither popular nor trending recently
- Other...

Topic: Digital Mental Health Intervention
Research in this topic area is characterised by words such as "digital, relatives, psychosis, user, carers, videos, react, users, platform, implementation, engagement, recovery, online, website, fep". An exemplary recent publication in this topic area is:
Fatouros et al. *Randomized controlled study of a digital data driven intervention for depressive and generalized anxiety symptoms*
https://pubmed.ncbi.nlm.nih.gov/39972054/

- Trending recently
- Popular but not specifically trending recently
- Neither popular nor trending recently
- Other...

Thank you for completing our survey! Do you have any questions or comments, feedback or suggestions for our research? Please enter them here.
